# Psychological and cognitive support after cardiac arrest: a systematic review

**DOI:** 10.1016/j.resplu.2026.101450

**Published:** 2026-08-15

**Authors:** Giuseppe Ristagno, Erga Cerchiari, Fabiana Madotto, Alberto Cucino, Aurora Magliocca, Giulia Merigo, Riccardo Boverio, Katya Ranzato, Andrea Scapigliati, Federico Semeraro, Lorenzo Gamberini, Fondazione Italian Resuscitation Council

**Affiliations:** a S.C. Ricerca Innovazione e Trasferimento Tecnologico, ASST Grande Ospedale Metropolitano Niguarda, Milan, Italy; b Fondazione Italian Resuscitation Council – ETS, Bologna, Italy; c Fondazione IRCCS Ca’ Granda Ospedale Maggiore Policlinico, Milan, Italy; d Department of Anaesthesia and Intensive Care Medicine, APSS Trento, Provincia Autonoma di Trento, Italy; e Agenzia Regionale Emergenza Urgenza – AREU, Milan, Italy; f Department of Department of Pathophysiology and Transplantation, University of Milan, Italy; g Department of Emergency Medicine, Azienda Ospedaliero-Universitaria SS. Antonio e Biagio e Cesare Arrigo, Alessandria, Italy; h Italian Resuscitation Council, Bologna, Italy; i Department of Cardiovascular Sciences, Cardiac Anaesthesia and Intensive Care Unit, Fondazione Policlinico Universitario “A. Gemelli” IRCCS, Rome, Italy; j Department of Anaesthesia, Intensive Care and EMS, Maggiore Hospital, Bologna, Italy

**Keywords:** Psychological support, Cognitive support, Survivors, Cardiac arrest

## Abstract

**Background:**

Adult survivors of cardiac arrest (CA) with favourable neurological recovery often experience persistent cognitive and psychological difficulties that impact quality of life and functional independence. However, post-discharge interventions remain heterogeneous, and evidence to support the prioritisation and implementation of specific cognitive and psychological interventions is limited.

**Objective:**

To systematically review the evidence on psychological and cognitive support interventions delivered after hospital discharge in adult CA survivors with good neurological recovery.

**Methods:**

A systematic review was conducted following PRISMA 2020 guidelines (PROSPERO CRD420251267160). Eligible studies included adults surviving CA with favourable neurological recovery who received structured post-discharge interventions. Outcomes were assessed across multiple domains, including: psychological well-being; cognitive performance; functional status and participation; health-related and global quality of life; and feasibility and implementation.

**Results:**

Six studies (*n* = 352 survivors) were included: two randomised controlled trials, two feasibility/pre-post studies, one single-case experimental design, and one process evaluation. Interventions varied in content and delivery (face-to-face, remote, home-based) and included nurse-led follow-up, psychotherapy, cognitive rehabilitation, and mindfulness-based strategies. Small improvements were observed in anxiety, depression, post-traumatic stress symptoms, and selected health-related quality-of-life domains, whereas cognitive, functional, and participation in daily and social activities showed limited or short-term gains in small uncontrolled studies. Interventions were feasible and well-tolerated.

**Conclusions:**

Post-discharge psychological and cognitive interventions are feasible and associated with modest improvements in psychological well-being and selected quality-of-life domains. Further rigorous research using standardised outcomes is needed.

## Introduction

Survival after cardiac arrest (CA) has increased over the past two decades due to improvements in the chain of survival, resuscitation interventions and techniques, and post-resuscitation care.^1^, ^2^ Nevertheless, even among survivors discharged with favourable neurological recovery, persistent cognitive, psychological, and emotional difficulties are common.^2^ These impairments may adversely affect long-term well-being and limit social reintegration.^3^, ^4^, ^5^ Indeed, survivors frequently report deficits in memory, attention, and executive functioning, as well as symptoms of anxiety, depression, post-traumatic stress, chronic fatigue, and reduced participation in daily activities.^6^, ^7^, ^8^, ^9^ Such sequelae may persist for months or even years after hospital discharge, compromising quality of life, increasing key supporter burden, and potentially influencing long-term clinical outcomes.^10^, ^11^

Despite growing recognition of the neuropsychological components of recovery after CA, post-discharge care remains highly variable. Cardiological and physical rehabilitation follow-up pathways are more established, whereas structured psychological and cognitive support is less consistently integrated.^2^, ^12^, ^13^ In addition, studies evaluating these interventions are heterogeneous in terms of design, intervention content and delivery, theoretical approach, and outcome measures, making it difficult to draw robust and generalizable conclusions.^14^, ^15^, ^16^

The European Resuscitation Council 2025 guidelines emphasise the importance of screening for neuropsychological problems and providing multidisciplinary follow-up, including psychological support when needed.^2^ However, while guidelines clearly recognise psychological morbidity as a key component of survivorship, recommendations remain relatively non-specific in terms of interventions to be implemented, at what intensity, or with what expected outcomes.^2^, ^6^ This is particularly relevant because the implementation of structured cognitive and psychological support pathways requires evidence demonstrating not only the presence of unmet needs, but also the capability of targeted interventions to improve patient-centred outcomes. In addition, the evidence base for post-discharge psychological and cognitive interventions remains limited. Most available studies are small, single-centre, or exploratory, with only a few randomised controlled trials (RCTs) conducted. Comparative effectiveness research is scarce, long-term outcomes are insufficiently evaluated, and no prior systematic review has comprehensively assessed psychological and cognitive support specifically in survivors with good neurological recovery after hospital discharge, a population whose rehabilitation needs may be underestimated.

Addressing the above knowledge gap is essential to inform clinical practise, support the prioritisation and development of structured cognitive and psychological support and follow-up pathways, and identify priorities for future research. Thus, the objective of this systematic review is to evaluate the effectiveness of psychological and cognitive support interventions delivered after hospital discharge in adult CA survivors with favourable neurological recovery. Primary outcomes include quality of life, psychological well-being, cognitive performance, functional status, and participation. Through rigorous synthesis of the available evidence, this review aims to provide a comprehensive foundation for improving post-CA care and informing future research directions.

## Methods

This systematic review was conducted in accordance with the PRISMA 2020 and the Synthesis Without Meta-analysis (SWiM) reporting guidelines.^17^ The protocol was prospectively registered in PROSPERO (CRD420251267160), and the review question was defined a priori using the PICO framework.

### PICO framework

#### Population

Adults (≥18 years) who survived in-hospital or out-of-hospital CA and were discharged with favourable neurological recovery as defined in each original study, including Cerebral Performance Category (CPC) 1–2 or equivalent (i.e. Modified Rankin Scale (mRS) 0–2, and the Glasgow Outcome Scale – Extended (GOSE) 7–8) when reported, independent living, intact cognition, or no severe cognitive impairment.

#### Intervention

Structured psychological, cognitive, or neuropsychological support delivered after hospital discharge, including cognitive rehabilitation, psychotherapeutic interventions, mindfulness-based programmes, psychoeducational approaches, or multidisciplinary follow-up pathways with psychological or cognitive components.

#### Comparator

Usual care, no intervention, wait-list control, or alternative psychological or cognitive interventions.

#### Outcomes

Primary outcomes were quality of life, cognitive performance, psychological symptoms (anxiety, depression, post-traumatic stress), emotional well-being, fatigue, and functional status. Secondary outcomes included key supporter burden, satisfaction with care, and other patient-reported measures related to psychological or cognitive recovery. Feasibility and implementation outcomes were considered as additional outcomes when reported. Outcomes were assessed at the longest follow-up available.

### Outcome assessment rationale

Primary outcomes were selected to reflect the core domains most consistently affected in adult CA survivors with favourable neurological recovery, in line with ERC 2025 recommendations.^2^ Guidelines highlight that long‑term recovery is multidimensional, with persistent difficulties commonly reported across cognitive, emotional, fatigue‑related, physical, and participation domains; condition‑specific measures are therefore recommended to capture this breadth. Secondary outcomes were conceptually related but classified separately because they refer to key supporters rather than survivors or represent complementary aspects of recovery that are not consistently measured across studies and are less directly comparable. This distinction also facilitated the organisation of the narrative synthesis, in accordance with PRISMA and SWiM guidance.

### Eligibility criteria

Eligibility criteria were defined a priori in the PROSPERO protocol and operationalised the PICO framework.

#### Inclusion criteria

Studies that: enrolled adult CA survivors with favourable neurological recovery; evaluated a defined post-discharge psychological or cognitive intervention; reported at least one of the outcomes of interest; used a randomised, quasi-experimental, controlled cohort, or observational design with a comparator; were published in English in peer-reviewed journals.

#### Exclusion criteria

Studies that: involved paediatric populations or adults with severe neurological impairment without a separate reporting for survivors with favourable recovery; evaluated interventions delivered exclusively during the index hospitalisation; were case reports, case series, cross sectional studies, conference abstracts, reviews, editorials, or qualitative studies without quantitative outcomes; did not report any psychological, cognitive, emotional, or functional outcome.

### Information sources and search strategy

A comprehensive literature search was conducted in MEDLINE (via PubMed), Embase, and Cochrane Central Register of Controlled Trials (CENTRAL), from database inception to December 31st, 2025. The search strategy was initially developed in MEDLINE and subsequently adapted to the syntax and controlled vocabulary of Embase and CENTRAL. The full search strategies for all databases are presented in the Supplemental Table 1.

### Study selection

All retrieved records were imported into a reference management system Rayyan software (Qatar Computing Research Institute, Doha)^18^ and duplicates were removed. Two authors (AC, GR) independently screened titles and abstracts, followed by full-text assessment of potentially eligible studies. Disagreements were resolved through discussion or consultation with a third author (LG). Reasons for exclusion at the full-text stage were documented.

### Data extraction

Two authors (AC, GR) independently extracted data using a standardised and piloted data extraction form. Extracted variables included study design, setting, sample size, participant characteristics, intervention and comparator details, outcome measures, follow-up duration, and effect estimates.

### Risk of bias assessment

Risk of bias was assessed independently by two authors (AC, GR). For RCTs, the RoB 2 tool^19^ was used, while for non-randomised studies, both the Risk of Bias in N-of-1 Trials (RoBiNT) tool^20^ and the NIH Risk of Bias scale^21^ were applied according to the study type. Any discrepancies between reviewers were resolved by consensus. Judgments were summarised both narratively and visually, providing a clear overview of study quality and potential limitations.

### Data synthesis

Studies were grouped by outcome domain and, where relevant, by study design. Because effect measures and outcome instruments were not sufficiently comparable, findings were synthesised narratively using direction of effect and contextual interpretation based on study design, sample size, and risk of bias, rather than statistical pooling. Interventions were also grouped inductively after data extraction according to their main target domain (e.g. psychological, cognitive). Due to limited aggregable outcome measures, reported metrics were not transformed and results were shown as originally presented in the studies. Consequently, patterns and direction of findings were summarised qualitatively to identify similarities and differences across studies.

### Missing evidence

As no meta-analysis was conducted, formal quantitative assessments of missing evidence (e.g., funnel plots or small-study effect analyses) were not appropriate. Potential missing evidence was examined by reviewing study registrations and comparing pre-specified outcomes with those reported in the published articles.

### Confidence of evidence

Two authors (AC, GR) independently assessed the certainty of evidence using the GRADE approach adapted for narrative syntheses.^22^ For each outcome, we evaluated risk of bias, inconsistency, indirectness, imprecision, and, when applicable, publication bias, following guidance for applying GRADE without a pooled effect estimate. Certainty was rated as High, Moderate, Low, or Very Low based on the overall confidence that the observed effects reflect the true effect. Discrepancies were resolved by consensus.

## Results

### Study selection

**Fig. 1:**
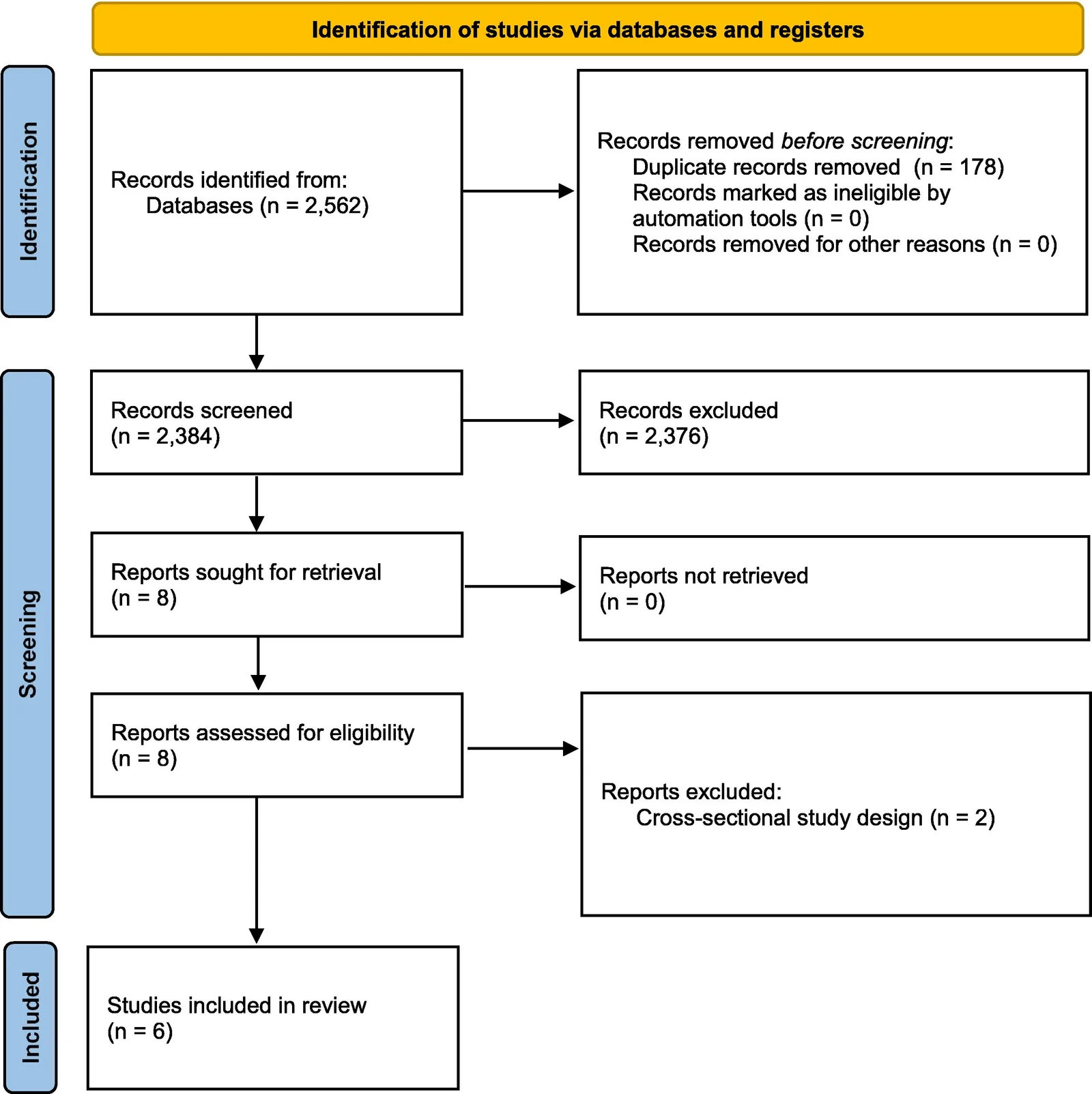
**PRISMA flow diagram**. The diagram summarises the identification, screening, and eligibility assessment of records retrieved through database searches. Of the 2384 records screened, eight full-text reports were evaluated for eligibility.

A total of 2562 records were identified through database searching. After removal of duplicates (*n* = 178), 2384 records were screened by title and abstract. Eight full-text articles were assessed for eligibility. Two articles were excluded due to a cross-sectional observational design. Six studies were included in the final synthesis, including one process evaluation conducted alongside an RCT, which contributed feasibility and implementation data but not comparative effectiveness outcomes. The study selection process is summarised in the PRISMA flow diagram (Fig. 1).

### Study characteristics

The six included studies^8^, ^14^, ^15^, ^23^, ^24^, ^25^ in terms of study design, intervention setting, sample size, population characteristics, and type of CA, reflected the diversity of approaches to post-discharge psychological and cognitive support for CA survivors, as summarised in Table 1.

**Table 1:** Summary of study design, setting, and participant characteristics.

**Study**	**Study design**	**Country**	**Year of enrolment**	**Intervention setting**	**Sample size** **(*n*)**	**Type of CA**	**Age** **(mean ± SD)**	**Females** **(%)**	**Neurological status at enrolment**	**Time from CA to enrolment**
Moulaert et al.^14^	Multicentre RCT	Netherlands	2007–2010	Hospital-, outpatient- and home-based with optional telephone follow-up	185 *(97 treatment, 88 control)* + 155 key supporters *(91 treatment, 64 control)*	IHCA, OHCA	≥18 y-o (60 ± 12)	17	NA (life expectancy >3 months)	∼2 weeks
Moulaert et al.^23^	Feasibility study alongside RCT	Netherlands	2007–2010	Hospital-, outpatient- and home-based with optional telephone follow-up	97 + 91 key supporters	IHCA, OHCA	≥18 y-o (60 ± 12)	17	NA (life expectancy >3 months)	∼2 weeks
Cowan et al.^24^	RCT	United States	<1999	Outpatient clinic-based	133 *(67 treatment, 66 control)*	OHCA	≥18 y-o (NA)	27	NA (no life-threatening comorbidities)	Mean ± SD − 21 ± 15 months in intervention group − 15 ± 13 months in comparator group
Kim et al.^8^	Pre-post feasibility study	United States	2010–2014	Telephone-based	18	IHCA, OHCA	≥18 y-o (53 ± 11)	44	Intact cognition	Time from CA to baseline: 96–2518 days (range), 114 days (median)
Bergman et al.^15^	Pre-post feasibility study	United States	2021–2022	Remote individual	11	IHCA, OHCA	≥18 y-o (52 ± 9)	64	No severe cognitive impairment (MMSE ≥ 23)	Time from CA to intervention (mean ± SD): 30 ± 21 months
van Gils et al.^25^	SCED non-concurrent multiple baseline	Netherlands	2022–2024	Home-based with remote assessment	5	OHCA	≥18 y-o (59 ± 9)	0	Good functional recovery Living independently Physician-indicated cognitive rehabilitation	Time from CA to intervention (mean ± SD): 11 ± 4 months

CA, cardiac arrest; IHCA, in-hospital CA; MME, mini-mental state examination; NA, not available; OHCA, out-of-hospital CA; RCT, randomised controlled trial; SCED, single-case experimental design; SD, standard deviation.

### Study design and setting

The review included two RCTs,^14^, ^24^ two single-arm feasibility/pre-post studies,^8^, ^15^ one single-case experimental design (SCED) with multiple baseline,^25^ and one feasibility/process evaluation^23^ conducted alongside one of the included RCTs, the Activity and Life After Survival of a Cardiac Arrest trial (ALASCA).^14^ This study focused exclusively on the experimental group and provided data on intervention acceptability and implementation, without contributing comparative effectiveness outcomes, and was therefore not included in the descriptive synthesis of the study results. Only the two RCTs^14^, ^24^ included a comparator group consisting of usual care, while the remaining studies adopted uncontrolled interventional designs (Table 2).

**Table 2:** Characteristics of psychological and cognitive interventions delivered to cardiac arrest survivors in the included studies.

**Study**	**Comparator**	**Intervention**								
		**Type**	**Approach**	**Delivery mode**	**Target participant**	**Format**	**Duration of sessions**	**Key components**	**At-home practise**	**Provider training**
Moulaert et al.^14^ Moulaert et al.^23^	Standard of care –	Individualised nurse-led post-discharge follow-up	– Early detection of cognitive, emotional problems – Self-management – Supportive care	– Face-to-face – Optional phone	– Patient – Key supporter	Individual	1–6 sessions, 30–60 min each	Screening for cognitive, emotional problems, information provision, self-management strategies, referral to specialised care	– Optional telephone follow-up; – home application of self-management strategies	Trained nurses, trained in intervention protocol and assessment instruments

Cowan et al.^24^	Standard of care	Psychosocial therapy	– CBT for depression, anxiety, anger – Physiologic relaxation with biofeedback – Health education	Face-to-face	Patient	Individual	11 biweekly sessions, 90 min each	Relaxation & biofeedback training, cognitive-behavioural therapy, cardiovascular risk education	Cognitive exercises for autonomic regulation monitored via biofeedback	Nurses with master’s degree in psychosocial nursing, specialised training in intervention protocol

Kim et al.^8^	−	Energy Conservation + Problem-Solving Therapy (EC + PST)	– Energy conservation for fatigue management; – PST framework	Telephone-based	Patient	Individual	Up to 8 sessions, 45 min each, twice weekly	Identify fatigue-related problems, generate solutions, implement action plans, review & adjust	Daily implementation of problem-solving strategies with participant workbook	Investigator trained in EC + PST, experience in fatigue management

Bergman et al.^15^	−	AMBET	– Acceptance & mindfulness-based interventions; – CBT principles for PTSD and emotion regulation	Teletherapy (video sessions, Zoom)	Patient	Individual	8 weekly sessions, 90 min each	Psychoeducation, mindfulness training, in vivo & imaginal exposure, trauma narrative	Weekly home practise assigned, worksheets provided	Two master’s-level clinicians with extensive experience in PTSD therapy; supervised by experienced psychiatrist

van Gils et al.^25^	−	Cognitive rehabilitation with metacognitive strategy training	– Metacognitive strategy + direct cognitive function training; – Evidence-based clinical practise	Outpatient clinic + home-based computer training	Patient	Individual	– 8–10 weekly-biweekly sessions, 1 h each; – 15–20 min/day computer training at home, 5 days/week	Strategy instruction, daily life application, cognitive exercises, therapist supervision	Computer training at home, reflection on strategy use, adherence monitored	Cognitive rehabilitation therapists or occupational therapists trained in protocol; adherence monitoring

CBT, cognitive-behavioural therapy; EC, energy conservation; PST, problem-solving therapy; AMBET = acceptance and mindfulness-based exposure therapy; PTSD, post-traumatic stress disorder.

Studies were conducted in the United States (*n* = 3)^8^, ^15^, ^24^ and the Netherlands (*n* = 2),^14^, ^25^ in intervention settings including: hospital- and home-based care with optional telephone follow-up^14^; outpatient clinic-based care^24^; telephone-based intervention^8^; remote individual intervention^15^; and home-based intervention with remote assessment^25^ (Table 1).

Enrolment periods spanned from the late 1990s to 2024.

### Population characteristics

**Fig. 2:**
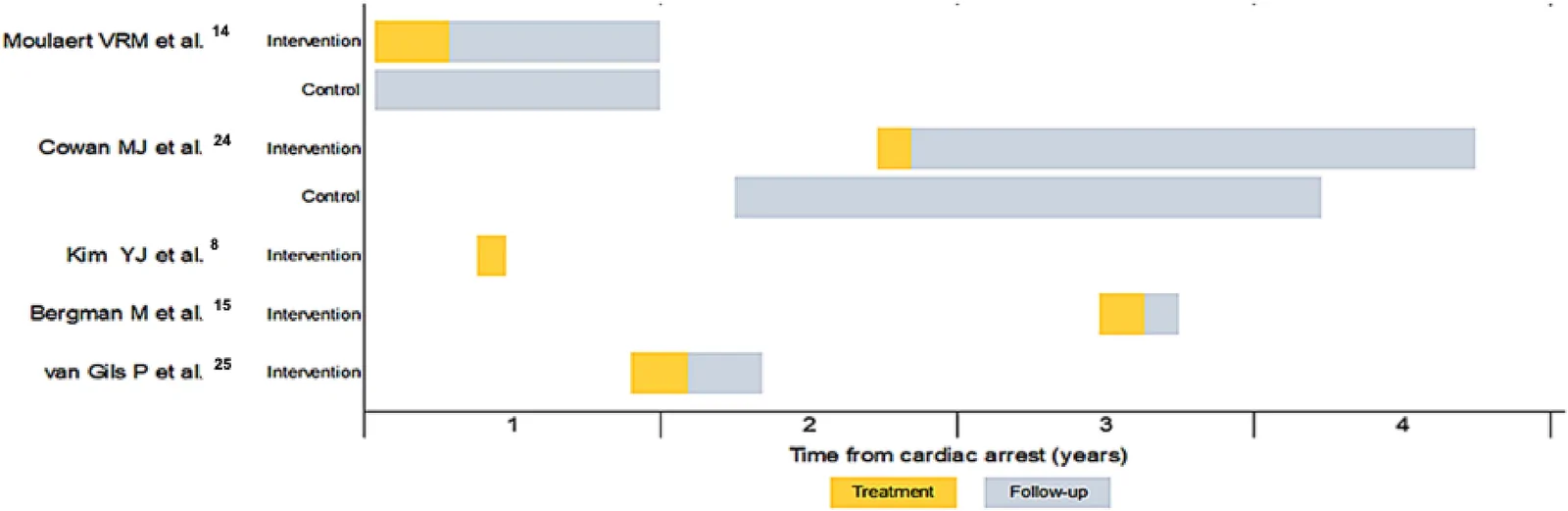
**Overview of intervention timing and follow-up duration**. The figure shows the heterogeneity in intervention initiation, treatment duration, and follow-up across included studies. Interventions were started as early as 2 weeks post-cardiac arrest or implemented several years later. Follow-up periods varied from short-term assessments to up to two years after the intervention. Treatment duration, follow-up length, and time from cardiac arrest to intervention are represented using the meantime points reported in each study. One study^23^ was not included in the figure as it was a process evaluation conducted alongside the ALASCA trial.^14^

Across all studies, a total of 352 adult CA survivors were analysed. Mean age ranged from 52 to 60 years, and the proportion of female participants ranged from 0% to 64%. Both in-hospital and out-of-hospital CAs were represented. Participants had good functional or neurological recovery according to study-specific inclusion criteria, which ranged from intact cognition and independent living to physician-indicated cognitive rehabilitation, although formal definitions varied. Time from CA to enrolment or intervention varied widely across studies, ranging from approximately 2 weeks up to 30 months (Fig. 2).

### Intervention characteristics

A wide range of intervention approaches was evaluated, differing in content, theoretical approach, intensity, delivery format, and timing, as summarised in Table 2.

Three broad categories were identified: *cognitive and neuropsychological rehabilitation programmes*, focusing on metacognitive strategy training and direct cognitive exercises, and delivered in outpatient settings with structured home-based computerised practice^25^; *psychological and psychotherapeutic interventions*, targeting emotional and behavioural sequelae of CA, including anxiety, post-traumatic stress symptoms, and fatigue, and grounded in cognitive-behavioural, problem-solving, acceptance-based, and mindfulness-based approaches^8^, ^15^, ^24^; *structured nurse-led follow-up programmes*, aiming at early detection of cognitive and emotional problems, provision of information and support, promotion of self-management, and referral to specialised care.^14^, ^23^

All interventions were delivered in an individual format. Delivery modalities included face-to-face sessions, telephone-based interventions, video-based teletherapy, and hybrid models combining clinic-based sessions with home practise. The number of sessions ranged from one to eleven, with session duration varying between 30 and 90 min. Interventions were delivered by trained healthcare professionals, including nurses, occupational therapists, cognitive rehabilitation therapists, and licenced mental health clinicians.

### Outcomes assessed

A wide range of psychological, cognitive, functional, behavioural, cardiovascular, and health-related outcomes following CA was assessed. A detailed overview of all outcome domains and corresponding assessment tools is provided in Table 3.

**Table 3:** Outcome domains and measurement instruments used across included studies.

**Outcome domain**	**Construct assessed**	**Instrument**	**Description/focus**	**Study**	**Primary outcome**
**A. Psychological and emotional outcomes**					
	Post-traumatic stress	Clinician-Administered PTSD Scale for DSM-5 (CAPS-5)	Clinician-rated PTSD symptom severity	15	No
		Posttraumatic Stress Disorder Checklist (PCL-5)	Self-reported PTSD symptoms	15	No
		Impact of Event Scale (IES)	Subjective distress related to traumatic events	14	No
	Depression	Hamilton Depression Rating Scale (HAM-D)	Clinician-rated depressive severity	15	No
		Beck Depression Inventory-II (BDI-II)	Self-reported depressive symptoms	15	No
	Anxiety and depression	Hospital Anxiety and Depression Scale (HADS)	Anxiety and depression screening	14	No
	General psychological distress	Symptom Checklist-90-Revised (SCL-90-R)	Broad psychological symptom profile	24	No
	Cardiac-related anxiety	Anxiety Sensitivity Index-3 (ASI-3)	Fear of anxiety-related bodily sensations	15	No
	Interoceptive awareness	Multidimensional Assessment of Interoceptive Awareness (MAIA-2)	Awareness of bodily sensations	15	No

**B. Cognitive outcomes**					
	Orientation, memory, executive function	Cognitive Log (Cog-Log)	Global cognitive screening	14	No
	Memory, processing speed	Adult Memory and Information Processing Battery (AMIPB)	Memory and information processing	14	No
	Attention, processing speed, executive function	Trail Making Test A/B (TMT-A/B)	Visual attention and task switching	14	No
	Executive function	Verbal Fluency	Phonemic word generation and executive control	14	No
		Letter Fluency	Phonemic word generation and executive control	25	No
	Verbal memory	Paragraph Recall (immediate/delayed)	Short- and long-term verbal memory	14	No
	Everyday memory failures	Everyday Memory Questionnaire-Revised (EMQ-R)	Subjective everyday memory problems	25	No
	Attention/working memory	Digit Span	Attention and working memory capacity	25	No
	Memory	Rey Auditory Verbal Learning Test (RAVLT)	Verbal learning and memory	25	No
	Subjective cognitive complaints	Cognitive Complaints Inventory/Interview (CLC-IC)	Perceived cognitive difficulties	25	No
	Cognitive lapses	Cognitive Failures Questionnaire (CFQ)	Frequency of everyday cognitive slips	14	No
	Daily cognitive problems	Visual Analogue Scales (VAS)	Individualised daily symptom tracking	25	YES

**C. Functional status and participation**					
	Self-care and daily activities	Performance Assessment of Self-Care Skills Recall (PASS-SR)	Functional task performance	8	No
	Instrumental activities of daily living	Functional Activities Questionnaire (FAQ)	IADL functioning	8	No
	Participation restrictions	Utrecht Scale for Evaluation of Rehabilitation-Participation (USER-P)	Societal participation	25	No
	Extended daily activities	Frenchay Activities Index (FAI)	Complex daily activities	14	No
	Community participation	Community Integration Questionnaire (CIQ)	Social and community integration	14	YES
	Objective and subjective participation	Participation Objective, Participation Subjective (POPS)	Self-report (objective and subjective participation domains)	8	No
	Vocational reintegration	Return to work	Descriptive employment outcome	14	No
	Key supporter burden	Caregiver Strain Index (CSI)	Key supporter stress and burden	14	No

**D. Fatigue**					
	Fatigue impact	Modified Fatigue Impact Scale (MFIS)	Impact of fatigue on daily life	8	No
	Fatigue severity	Fatigue Severity Scale (FSS)	Fatigue severity	8	No
	Patient-reported fatigue	PROMIS Fatigue Scale	Patient-reported fatigue	8	No

**E. Health-related and global quality of life**					
	Health-related quality of life	Short Form-36 Health Survey (SF-36)	Multidimensional HRQoL	14	YES
	Global perceived health	EuroQol Visual Analogue Scale (EQ-VAS)	Overall self-rated health	14	YES
	Global life satisfaction	Life Satisfaction Questionnaire (LiSat-9)	Overall life satisfaction	25	No

**F. Cardiovascular clinical endpoints**					
	Mortality	CV and all-cause mortality	Survival outcomes	24	CV: YES All-cause: No
	Cardiac events	Non-fatal cardiac events	MI, arrhythmias, device firings	24	No

**G. Behavioural and physiological markers**					
	Physical activity (daily movement)	Daily steps	Objective (device-based)	15	No
	Sedentary behaviour	Sedentary time	Objective (device-based)	15	No
	Sleep patterns	Sleep duration	Objective (device-based)	15	No
	Autonomic regulation	Heart rate variability	Exploratory physiological marker	24	No
		Heart rate	Cardiovascular regulation	24	No
		Breathing rate	Respiratory pattern	24	No

**H. Feasibility and implementation outcomes**					
	Satisfaction	Client Satisfaction Questionnaire (CSQ-8/CSQ-3)	Intervention satisfaction	8, 15	YES
	Understanding of intervention materials	Understanding of Materials Scale (UMS)	Comprehension and perceived clarity of intervention materials	8	YES
	Implementation metrics	Recruitment, retention, adherence, fidelity	Feasibility indicators	8, 15, 23–25	8, 15, 23: YES 24, 25: No

CV, cardiovascular.

*Psychological outcomes* were assessed in three studies.^14^, ^15^, ^24^ These included post-traumatic stress symptoms, depression, anxiety, cardiac-related anxiety, general psychological distress, and interoceptive awareness, measured through a combination of clinician-administered and self-report instruments.

*Cognitive outcomes* were evaluated in two studies,^14^, ^25^ using both objective neuropsychological assessments targeting memory, attention, processing speed, and executive functioning, as well as subjective reports of cognitive complaints and everyday cognitive failures.

*Functional status and participation outcomes* were reported in three studies,^8^, ^14^, ^25^ including measures of activities of daily living, instrumental activities, community participation, vocational reintegration, and key supporter burden.

*Fatigue* was assessed as a distinct symptom domain in one study.^8^

*Health-related quality of life (HRQoL)* and *global well-being* were examined in two studies,^14^, ^25^ using multidimensional quality-of-life instruments and life satisfaction measures.

*Cardiovascular clinical endpoints*, including mortality and recurrent cardiac events, were reported in one study.^24^

*Behavioural and physiological markers*, such as physical activity, sleep patterns, and autonomic regulation markers, were included in two studies.^15^, ^24^

Finally, *feasibility and implementation outcomes* (including recruitment, retention, adherence, intervention fidelity, and participant satisfaction) were reported in all studies,^14^, ^15^, ^23^, ^24^, ^25^ primarily as process indicators rather than clinical endpoints.

Follow-up durations varied across studies, ranging from short-term assessments immediately after the intervention to longer-term evaluations, extending up to two years (Fig. 2).

### Intervention effects across studies

Controlled trials assessed intervention effects relative to standard care or cardiology follow-up, whereas single-case and feasibility studies evaluated within-subject changes over time. Across studies, intervention effects were reported in multiple outcome domains, as summarised in Table 4 and Supplemental Table 2 and Supplemental Table 3.

**Table 4:** Primary outcomes and observed effects across studies.

**Study**	**Follow-up**	**Outcome domain**	**Effect/Direction of change**
**Controlled studies**			
Moulaert et al.^14^	1 year after CA*	[C] Community participation	No change
		[E] Health-related quality of life	Improvement in some domains (role emotional, mental health, bodily pain, general health)
		[E] Global perceived health	No change
Cowan et al.^24^	2 years after enrolment	[F] Cardiovascular mortality	Decrease

**Not controlled studies**			
Moulaert et al.^23^	Post-treatment (∼3 months)	[H] Feasibility/Acceptability	High attendance: sessions delivered as planned; positive satisfaction reported by patients and key supporters
Kim et al.^8^	Post-treatment (4 weeks)	[H] Feasibility/Acceptability	High retention and adherence; intervention delivered as planned via telephone; high participant satisfaction and understanding of materials
Bergman et al.^15^	Post-treatment (8 weeks), 3 months	[H] Feasibility/Acceptability	High adherence and fidelity; no adverse events; high satisfaction
van Gils et al.^25^	Post-treatment (6–10 weeks), 150 days	[B] Daily cognitive problems	Small improvement; not significant at group level

The square bracket reports the domain to which each outcome belongs, as specified in this table: [B] = Cognitive outcomes; [C] = Functional status and participation; [E] = Health-related and global quality of life; [F] = cardiovascular clinical endpoints; [H] = Feasibility and implementation outcomes.

* All outcomes were also evaluated at 3 months and no changes were observed.

#### Psychological outcomes

Three studies examined psychological outcomes as secondary endpoints.^14^, ^15^, ^24^ Two RCTs compared psychosocial interventions with standard care. Particularly, in the ALASCA trial,^14^ an early nurse-led intervention led to minor improvements in anxiety and depression at 12 months, but post-traumatic stress was unchanged. Cowan et al.^24^ tested a late psychosocial intervention, delivered on average 21 months after CA, with no significant improvement in psychological distress over two years versus standard care. In an uncontrolled pre-post feasibility study,^15^ participants showed reduced cardiac-related anxiety and better interoceptive awareness after psychotherapy-based treatment. Depression and post-traumatic stress symptoms improved further at 3-month follow-up.

#### Cognitive outcomes

Cognitive outcomes were evaluated in one RCT^14^ and one SCED study,^25^ each with different follow-up periods. In the RCT, cognitive function was a secondary endpoint measured at 3 and 12 months using a broad neuropsychological battery; no significant group differences emerged. The SCED study, with shorter follow-up (6–10 weeks post-treatment, up to 150 days), tracked daily cognitive problems as the primary outcome and found a slight reduction over time without statistical significance. Exploratory endpoints showed minor within-subject improvements during the intervention.

#### Functional status and participation outcomes

One RCT^14^ assessed functional and participation outcomes as the primary endpoint, while two uncontrolled studies evaluated them as secondary outcomes.^8^, ^25^ The ALASCA trial^14^ found no significant differences between groups at 12 months, including extended daily activities and return to work. The feasibility study^8^ reported unchanged functional outcomes from baseline to 4 weeks, with some ceiling effects. In the SCED study,^25^ a minor reduction in participation restrictions was noted in certain participants during intervention. Overall, comparative evidence did not show improvements in functional status or participation; any observed improvements were limited to short-term changes within small uncontrolled samples.

#### Fatigue

Fatigue was evaluated as a secondary endpoint in an uncontrolled feasibility study^8^ after 4 weeks of treatment. The intervention reduced perceived fatigue's impact on daily life but did not consistently affect overall fatigue severity or patient-reported fatigue compared to baseline.

#### Health-related and global quality of life

Only one RCT^14^ assessed HRQoL as a primary outcome. After 12 months, nurse-led intervention survivors improved in select SF-36 domains (role emotional, mental health, bodily pain, general health) compared to standard care, with no difference in global perceived health (EQ-VAS). The SCED study^25^ measured global life satisfaction (LiSat-9) over a shorter period, finding no meaningful change during treatment.

Overall, only specific HRQoL domains showed improvement; global self-rated health and overall life satisfaction remained unchanged.

#### Cardiovascular clinical endpoints

Only one RCT^24^ evaluated cardiovascular endpoints two years after the intervention, reporting a reduction in cardiovascular mortality (primary outcome) in the intervention group, with no differences in all-cause mortality or non-fatal cardiac events (secondary outcomes) compared with standard care.

#### Behavioural and psychological markers

Two studies evaluated secondary behavioural and physiological outcomes: an RCT^24^ and a feasibility study.^15^ In the RCT, minor improvement in breathing rate was noted at 2-year follow-up, with no change in heart rate or variability. The feasibility study found increased daily steps and reduced sedentary time post-treatment (8 weeks), but sleep duration was unaffected.

#### Feasibility and implementation outcomes

Except for a RCT^24^ and the SCED,^25^ all studies focused primarily on feasibility, acceptability, and implementation. Interventions had high session adherence, good completion of home exercises, and strong participant satisfaction; they were delivered as planned in both individual and remote formats, with no major adverse events.

#### Key supporter outcomes

Key supporter outcomes were systematically evaluated in only one RCT.^14^ Although the intervention was associated with improved emotional well-being and quality of life among survivors at 12 months, it did not result in a significant reduction in key supporter strain compared with usual care.

### Risk of bias within studies

Risk of bias varied markedly across studies (Table 5). The ALASCA RCT^14^ showed low overall risk of bias and good internal validity, whereas Cowan et al.^24^ had high risk of bias due to unclear randomization, selective reporting concerns, and outdated post-cardiac arrest care.

**Table 5:** Summary of risk of bias across included studies.

**Study**	**Risk of bias tool**	**Key limitations**	**Overall risk of bias**
Moulaert et al.^14^	RoB2	Partial blinding; modest sample; attrition at 12 months; heterogeneous outcomes; some deviations from intended intervention	Low to moderate
Cowan et al.^24^	RoB2	High risk of bias; survivor and selection bias; outdated care; small number of events; unclear blinding	High
Kim et al.^8^	NIH Before–After Tool	Small sample; no control group; short follow-up; selected population; ceiling effects	Poor (high risk of bias)
Bergman et al.^15^	NIH Before–After Tool	Small, uncontrolled sample; no comparator; compensation may influence retention; short follow-up	Poor (high risk of bias)
van Gils et al.^25^	RoBiNT Scale	Very small sample; no blinding; baseline variability; limited generalizability; strong internal structure	Moderate

NIH, national institutes of health risk of bias assessment; RoB 2, risk of bias 2 tool; RoBiNT, risk of bias in *n*-of-1 trials scale.

The two single-arm feasibility studies^8^, ^15^ clearly described objectives, interventions, and outcomes, but were limited by small samples, poor representativeness, incomplete enrollment, and some methodological weaknesses in blinding and follow-up. These limitations reduced both internal validity and generalizability, leading to an overall poor-quality rating.

The single-case experimental design^25^ showed moderate internal validity, supported by randomised baselines and independent outcome assessment, with a clearly described and replicable intervention; however, it is very small, highly selected sample substantially restricted generalizability despite otherwise sound methodology.

The sixth included study,^23^ a process evaluation conducted alongside the ALASCA trial,^14^ was not formally evaluated for risk of bias in relation to intervention effects because it contributed feasibility and implementation data only, rather than comparative effectiveness outcomes.

### Missing evidence

Review of study registrations showed that all but one included study were prospectively registered, with primary and secondary reported outcomes consistent with those pre‑specified. One RCT^24^ lacked prospective registration, introducing minor uncertainty regarding selective outcome reporting.

### Confidence in evidence

Overall certainty ranged from low to very low across outcome domains, constrained by methodological limitations, small samples, and heterogeneity, with moderate certainty only for feasibility outcomes (Table 6). Psychological results were inconsistent, cognitive and functional effects were minimal, and improvements in other domains were limited or observed only in small uncontrolled studies. Key supporter outcomes remained largely unexamined. In outcome domains informed by the unregistered RCT,^24^ minor concerns about selective non‑reporting further contributed to the low certainty ratings.

**Table 6:** Confidence of evidence by outcome domain.

**Outcome domain**	**Effect**	**Participants (studies)**	Certainty in the evidence*
A. Psychological and emotional outcomes	Findings inconsistent across studies: small improvements in anxiety/depression in one RCT; no effect in another RCT; large improvements only in a very small uncontrolled study	329 (3 studies)	LOW ⊕⊕OO (due to serious risk of bias, inconsistency, imprecision and possible selective reporting)
B. Cognitive outcomes	No effect in RCT; small improvements in SCED	190 (2 studies)	LOW ⊕⊕OO (due to serious risk of bias, imprecision and possible selective reporting)
C. Functional status and participation	No effect in RCT; no effect and small improvements in small uncontrolled studies	208 (3 studies)	LOW ⊕⊕OO (due to serious risk of bias, inconsistency and possible selective reporting)
D. Fatigue	Small reduction in one small feasibility study	18 (1 study)	VERY LOW ⊕OOO (due to very serious risk of bias and very serious imprecision)
E. Health-related and global quality of life	Improvements in selected HRQoL domains; no change in global health	190 (2 studies)	LOW ⊕⊕OO (due to serious risk of bias and imprecision)
F. Cardiovascular clinical endpoints	Small reduction in CV mortality in one older RCT	133 (1 study)	LOW ⊕⊕OO (due to serious risk of bias and imprecision)
G. Behavioural and physiological markers	Large improvements in activity/sedentary time in one small uncontrolled study; small improvement in breathing rate in one RCT; no change in sleep, HR, or variability	144 (2 studies)	LOW ⊕⊕OO (due to serious risk of bias, inconsistency, imprecision, and possible selective reporting)
H. Feasibility and implementation outcomes	High adherence, fidelity, and acceptability across studies	198 treated (5 studies)	MODERATE ⊕⊕⊕O (limited by study design but consistent findings)

*Certainty of evidence was assessed using the GRADE approach: ⊕⊕⊕O = moderate certainty; ⊕⊕OO = low certainty; ⊕OOO = very low certainty.

## Discussion

Surviving a cardiac arrest is increasingly recognised as a life-changing transition that extends far beyond medical stabilisation, requiring a shared process of “negotiating a new normality” for both survivors and their key supporters.^26^, ^27^, ^28^, ^29^ This systematic review provides a focused overview of post-discharge psychological and cognitive support interventions for adult CA survivors with favourable neurological recovery and found that, while structured interventions show a potential favourable effect in alleviating psychological distress, significant gaps remain regarding cognitive and functional recovery.

Six studies were included,^8^, ^14^, ^15^, ^23^, ^24^, ^25^ among which only two were RCTs,^14^, ^24^ limiting the evidence base in both quantity and quality. Moreover, heterogeneity in design, intervention type, outcome measures, and follow-up duration precluded quantitative synthesis. Overall, across studies, small improvements were observed for anxiety, depression, and selected HRQoL domains, whereas cognitive, functional, and participation outcomes were less consistently affected and primarily showed short-term or within-subject benefits in small uncontrolled studies.

Overall, despite a weak signal towards potential improvements in some areas such as anxiety, depression, fatigue and HRQoL, the findings of this systematic review indicate that the current evidence base remains limited and fragmented, with substantial variation in intervention approaches, study designs, and outcome measures. Consequently, this immaturity of the field restricts the strength of any conclusions regarding intervention effectiveness. A previous systematic review by Joshi et al.^30^ similarly found insufficient evidence to determine whether rehabilitation interventions, considered broadly, improve quality of life, neurological recovery, or other functional outcomes in CA survivors. However, unlike that broader review,^30^ which examined rehabilitation interventions as a whole, this review narrows and updates the evidence base, focusing specifically on post-discharge interventions targeting the psychological and cognitive sequelae of survivorship.

### Psychological and psychotherapeutic interventions

The high prevalence of anxiety, depression, and PTSD reported across the literature underscores the need for the psychological support identified in this review.^31^, ^32^ Importantly, psychological distress is not merely a quality-of-life concern; it may also serve as a clinical warning signal, as post-CA depression and anxiety have been linked to substantially higher long-term mortality.^33^ In this context, the observed improvements associated with psychotherapy- and mindfulness-based approaches in this review are consistent with emerging evidence in the field. For example, the study by Bergman et al.^15^ has demonstrated feasibility in reducing PTSD symptoms, while dispositional mindfulness has been associated with lower psychological symptom burden and reduced severity of Enduring Somatic Threat (EST), reinforcing the rationale for integrating mind–body approaches into post-discharge programmes.^16^, ^34^

### Cognitive, neuropsychological rehabilitation and structured nurse-led follow-up programmes

Up to half of CA survivors face cognitive impairment, often accompanied by anxiety or depression, leading to social isolation and difficulty returning to work.^35^

While this review identified only limited or short-term improvements in cognitive outcomes, emerging evidence suggests that personalised, neurologically informed interventions are both feasible and potentially beneficial in post-cardiac arrest care.^23^, ^32^ In particular, the “Stand still …, and move on” nurse-led intervention^23^ showed that early identification of cognitive and psychological sequelae, combined with self-management support, can translate into sustained improvements in long-term quality of life. Similarly, small-scale studies indicate that interventions integrating metacognitive strategy training with direct cognitive training may reduce the burden of individualised cognitive difficulties encountered in daily life by survivors.^11^ However, the marked heterogeneity of post-cardiac arrest cognitive impairment, together with the frequent use of generic rather than condition-specific outcome measures, may attenuate the ability of larger trials to detect clinically meaningful benefits.^11^, ^30^

### Key supporters

A critical finding of this review is the striking paucity of key supporter outcome assessment, despite growing evidence that family members and other key supporters may experience psychological distress that is comparable to, or even greater than, that of survivors themselves.^26^, ^32^ Qualitative data suggest that key supporters endure substantial emotional turmoil and are often forced to navigate a “new and unknown world” as they assume a caregiving role. These experiences are not peripheral to recovery but deeply intertwined with survivor outcomes. Indeed, the observed association between survivor cognitive impairment and PTSD symptoms in relatives further supports the need for a family-centred model of rehabilitation.^36^ Importantly, current guidelines also extend the focus beyond survivors alone, recognising that key supporters often experience substantial emotional burden and should be incorporated into follow-up pathways and supportive care strategies.^2^, ^37^, ^38^, ^39^, ^40^, ^41^

### Outcome assessment instruments

Recent post-resuscitation guidelines have explicitly reframed survivorship after cardiac arrest as a multidimensional rehabilitation challenge rather than a purely cardiological follow-up issue.^2^, ^13^ This perspective strongly aligns with one of the central findings of our systematic review, i.e. the major limitation of the current literature lies not in the absence of clinically relevant sequelae, but in the fragmented way these outcomes are assessed. Recommendations for specific instruments to be used in multidimensional assessments in CA survivors further underscore the need for harmonised multidomain outcome measures in future trials.^42^, ^43^, ^44^, ^45^ Such harmonisation would improve the comparability and interpretability of findings across studies and facilitate evidence synthesis, while the use of validated and responsive measures appropriate for CA survivors may help distinguish clinically meaningful changes.

Although multidisciplinary follow-up, psychoeducation, cognitive rehabilitation, fatigue-targeted management, and referral to neuropsychological or rehabilitation specialists are all proposed as clinically reasonable strategies, much of this approach still relies on evidence extrapolated from acquired brain injury rehabilitation rather than on CA-specific trials.^2^, ^37^ This closely mirrors the findings of this review, where the available studies were largely exploratory and methodologically heterogeneous, with the most consistent signals of improvement emerging in psychological well-being and selected patient-reported outcomes rather than across broader functional domains. Taken together, these considerations strengthen the implication of our review that future trials should test integrated post-CA rehabilitation models using standardised multidomain endpoints and explicitly incorporating key supporter- or partner-oriented components, rather than evaluating isolated symptom-specific interventions alone.

### Limitations

Many studies had methodological limitations, such as small sample sizes, incomplete enrolment, lack of blinding, heterogeneous interventions, and varied follow-up durations, all of which weakened the evidence and limited its generalizability. Older trials further reduced relevance to current post-cardiac arrest care, resulting in low to very low certainty of evidence by modified GRADE assessment. Furthermore, the small number of eligible comparative studies reflects the current stage of this research field. Although standardised instruments are essential for quantifying intervention effects and enabling comparisons across studies, they may not fully capture the complex, relational, and evolving nature of recovery after cardiac arrest. Although qualitative and mixed-methods studies were not included in this review, their findings should complement quantitative outcome assessment by illuminating the lived experiences of survivors and key supporters, including how they perceive cognitive and psychological changes, adapt to altered roles and relationships, and experience the accessibility, relevance, and acceptability of support.

Nevertheless, this systematic review contributes uniquely by focusing on post-discharge psychological and cognitive support interventions for CA survivors, providing an evidence base to inform the prioritisation and implementation of specific and structured support programmes.

### Future directions

Future research should combine adequately powered effectiveness trials with qualitative, mixed-methods, and theory-driven approaches capable of capturing the contextual, relational, and experiential dimensions of post–cardiac arrest recovery. Moreover, the optimal timing, content, intensity, and delivery modalities of interventions, as well as the role and extent of key supporter involvement, remain to be established.^46^

## Conclusions

Post-discharge psychological and cognitive interventions for CA survivors with good neurological recovery are feasible and well-tolerated, with modest improvements in anxiety, depression, and select quality-of-life domains. Evidence for improvements in cognitive, functional, and key supporter outcomes is limited, and overall certainty is low due to methodological constraints and small, heterogeneous studies. Future research should include rigorous effectiveness trials alongside qualitative and mixed‑methods approaches to better capture the needs of this vulnerable population and to inform the development of integrated, survivor‑ and supporter‑centred rehabilitation pathways.

## Fundings

This study was funded by Fondazione Italian Resuscitation Council – ETS, Bologna, Italy.

## Data availability statement

The data extracted and analysed during this systematic review are available from the corresponding author upon reasonable request.

## Declaration of transparency/disclosure

Given her role as an Associate Editor, Aurora Magliocca had no involvement in the peer review of this article and had no access to information regarding its peer review. Full responsibility for the editorial process for this article was delegated to another journal editor.

## CRediT authorship contribution statement

**Giuseppe Ristagno:** Writing – original draft, Supervision, Investigation, Conceptualization. **Erga Cerchiari:** Writing – original draft, Funding acquisition, Conceptualization. **Fabiana Madotto:** Writing – original draft, Methodology, Data curation. **Alberto Cucino:** Writing – review & editing, Investigation, Data curation. **Aurora Magliocca:** Writing – review & editing, Investigation. **Giulia Merigo:** Writing – review & editing, Investigation. **Riccardo Boverio:** Writing – review & editing, Conceptualization. **Katya Ranzato:** Writing – review & editing, Conceptualization. **Andrea Scapigliati:** Writing – review & editing, Conceptualization. **Federico Semeraro:** Writing – review & editing, Conceptualization. **Lorenzo Gamberini:** Writing – review & editing, Data curation, Conceptualization.

## Declaration of competing interest

The authors declare the following financial interests/personal relationships which may be considered as potential competing interests: GR is member of the Editorial Board of Resuscitation Plus; AM is an Associate Editor of Resuscitation Plus; GR, FS, EC, RB are board members of Fondazione Italian Resuscitation Council – ETS; FM received a consultation fee from Fondazione Italian Resuscitation Council – ETS to lead the statistical methodology; LG is the principal investigator of ENFORCER trial, funded by Fondazione Italian Resuscitation Council – ETS, and an Associate Editor of the Scandinavian Journal of Trauma, Resuscitation and Emergency Medicine; AC, AM, LG are members of Italian Resuscitation Council (IRC) Scientific Committee; KR is the president of IRC; AS is past president of IRC; FS is the President of European Resuscitation Council and an Associate Editor of Artificial Intelligence in Emergency Medicine. GR is the Director Congresses of European Resuscitation Council. GM declares no conflicts.

## Appendix A Supplementary material

Supplementary Data 1 Supplementary tables providing the full search strategies across databases and detailed primary, secondary, and exploratory outcomes, including observed effect sizes and implementation outcomes across the included studies.
