# Supplementary material for: Psychological and cognitive support after cardiac arrest: a systematic review
Source: Resusc Plus. 2026 Aug 15;31:101450. doi: 10.1016/j.resplu.2026.101450 (PMC13559835; doi:10.1016/j.resplu.2026.101450)
Supplement: Supplementary file 1 [file mmc1.docx]

**Supplemental Table 1.** Full search strategies for all databases

| **Database** | **Search strategy** |
| --- | --- |
| **MEDLINE (PubMed)** | ("Heart Arrest"[MeSH Terms] OR "Cardiac Arrest"[All Fields] OR "Post-cardiac arrest"[All Fields] OR "Out-of-hospital cardiac arrest"[All Fields] OR "In-hospital cardiac arrest"[All Fields] OR "Heart Arrest"[All Fields] OR "cardiopulmonary arrest"[All Fields] OR "circulatory arrest"[All Fields] OR "Sudden Cardiac"[All Fields] OR "Post-resuscitation"[All Fields] OR "Return of spontaneous circulation"[All Fields] OR "Resuscitated cardiac"[All Fields] OR "resuscitation survivor*"[All Fields] OR "Post-ROSC"[All Fields] OR "ROSC"[All Fields] OR ("SCA"[All Fields] AND "cardiac"[All Fields]) OR "OHCA"[All Fields]) AND ("psycho* support*"[All Fields] OR "psycho* intervention*"[All Fields] OR "psycho* therap*"[All Fields] OR "psycho* care"[All Fields] OR "psycho* rehabilitation*"[All Fields] OR "Psychotherapy"[All Fields] OR ("Counseling"[All Fields] OR "Counselling"[All Fields]) OR ("cognitive behavioral therap*"[All Fields] OR "cognitive behavioural therap*"[All Fields]) OR "Mental Health Support"[All Fields] OR "Supportive Care"[All Fields] OR "Follow-up care"[All Fields] OR "Patient Education"[All Fields] OR "Psychotherapy"[MeSH Terms] OR "Counseling"[MeSH Terms] OR "psychosocial support systems"[MeSH Terms] OR "Health Education"[MeSH Terms] OR "Behavior Therapy"[MeSH Terms]) AND (1900/01/01:2025/12/31[Date - Publication]) |
| **Embase (Embase.com)** | ('heart arrest'/exp OR 'cardiac arrest':ti,ab OR 'post cardiac arrest':ti,ab OR 'out of hospital cardiac arrest':ti,ab OR 'in-hospital cardiac arrest':ti,ab OR 'heart arrest':ti,ab OR 'cardiopulmonary arrest':ti,ab OR 'circulatory arrest':ti,ab OR 'sudden cardiac':ti,ab OR 'post-resuscitation':ti,ab OR 'return of spontaneous circulation':ti,ab OR 'resuscitated cardiac':ti,ab OR 'resuscitation survivor*':ti,ab OR 'post-rosc':ti,ab OR 'rosc':ti,ab OR ('sca':ti,ab AND 'cardiac':ti,ab) OR 'ohca':ti,ab) AND ('psycho* support*':ti,ab OR 'psycho* intervention*':ti,ab OR 'psycho* therap*':ti,ab OR 'psycho* care*':ti,ab OR 'psycho* rehabilitation*':ti,ab OR 'psychotherapy*':ti,ab OR (counseling:ti,ab AND counselling:ti,ab) OR ('cognitive behavioral therap*':ti,ab OR 'cognitive behavioural therap*':ti,ab) OR 'mental health support':ti,ab OR 'supportive care':ti,ab OR 'follow-up care':ti,ab OR 'patient education':ti,ab OR 'psychotherapy'/exp OR 'psychological counseling'/exp OR 'psychosocial care'/exp OR 'health education'/exp OR 'behavior therapy'/exp) AND [01-01-1900]/sd NOT [01-01-2026]/sd AND [embase]/lim NOT ([embase]/lim AND [medline]/lim) |
| **Cochrane CENTRAL** | ([mh "Heart Arrest"] OR "Cardiac Arrest":ti,ab,kw OR "Post-cardiac arrest":ti,ab,kw OR "Out-of-hospital cardiac arrest":ti,ab,kw OR "In-hospital cardiac arrest":ti,ab,kw OR "heart arrest":ti,ab,kw OR "cardiopulmonary arrest":ti,ab,kw OR "circulation arrest":ti,ab,kw OR "sudden cardiac":ti,ab,kw OR "Post-resuscitation":ti,ab,kw OR "Return of spontaneous circulation":ti,ab,kw OR "Resuscitated cardiac":ti,ab,kw OR resuscitation NEXT survivor:ti,ab,kw OR "Post-ROSC":ti,ab,kw OR "ROSC":ti,ab,kw OR (SCA:ti,ab,kw AND cardiac:ti,ab,kw) OR OHCA:ti,ab,kw) AND (psycho NEXT support:ti,ab,kw OR psycho NEXT intervention:ti,ab,kw OR psycho NEXT therap:ti,ab,kw OR psycho NEXT care:ti,ab,kw OR psycho* NEXT rehabilitation:ti,ab,kw OR Psychotherapy:ti,ab,kw OR Counseling:ti,ab,kw OR Counselling:ti,ab,kw OR ("cognitive" NEXT "behavioral" NEXT therap:ti,ab,kw OR "cognitive" NEXT "behavioural" NEXT therap*:ti,ab,kw) OR "Mental Health Support":ti,ab,kw OR "Supportive care":ti,ab,kw OR "Follow-up care":ti,ab,kw OR "Patient education":ti,ab,kw OR [mh "Psychotherapy"] OR [mh "Counseling"] OR [mh "psychosocial support systems"] OR [mh "Health Education"] OR [mh "Behavior Therapy"])  *Limits: with Cochrane Library publication date from Jan 1900 to Jan 2026, (Word variations have been searched)* |

**Supplemental Table 2.** Primary outcomes and observed effect size across studies

| **Study** | **Follow-up** | **Outcome domain** | **Effect size** |
| --- | --- | --- | --- |
| **Controlled Studies** | | | |
| Moulaert VRM et al. (14) | 1 year after CA* | [C] Community participation | CIQ = mean difference ± SE = 1.19 ± 0.63 (p=0.062) |
|  |  | [E] Health-related quality of life | SF-36 Physical functioning = mean difference ± SE = 4.59 ± 3.71 (p=0.218) SF-36 social functioning = mean difference ± SE = 2.95 ± 3.61 (p=0.416) SF-36 role physical = mean difference ± SE = 7.95 ± 6.93 (p=0.253) SF-36 role emotional = mean difference ± SE = 16.38 ± 5.84 (p=0.006)  SF-36 mental health = mean difference ± SE = 6.87 ± 2.25 (p=0.003) SF-36 vitality = mean difference ± SE = 5.07 ± 2.70 (p=0.063) SF-36 bodily pain = mean difference ± SE = 9.46 ± 3.86 (p=0.016) SF-36 general health = mean difference ± SE = 8.07 ± 3.08 (p=0.010) |
|  |  | [E] Global perceived health | EQ-VAS = mean difference ± SE = 1.76 ± 2.31 (p=0.447) |
| Cowan MJ et al. (24) | 2 years after enrolment | [F] Cardiovascular mortality | Cardiovascular mortality = unadjusted HR (95%CI) = 0.14 (0.02-0.89) (p=0.03) |
| **Not Controlled Studies** | | | |
| Moulaert VRM et al. (23) | Post-treatment (~3 months) | [H] Feasibility / Acceptability | **Implementation outcomes** - Adoption / Uptake (attendance) = 81% received the intervention; 10% discontinued, 6% refused, 3% not treated for other reasons. - Data completeness (response rate) = 73% patient and 94% nurse response rate; nurses completed 96% of consultations. - Feasibility = 87% face-to-face and 13% telephone consultations; 1–5 sessions per patient; mean telephone duration 16 min; mean 18 min per consultation. 3 patients received only telephone consultations. - Acceptability (patients and caregivers) = 54% of patients and 56% of caregivers rated the intervention as useful; problems reported by 59% and 71%. >80% reported sufficient information, support, coping ability, and willingness to recommend; >70% considered timing appropriate. - Acceptability (nurses) = 95% rated intervention useful; all recommended implementation. Face-to-face delivery considered essential; home visits valued but time-consuming. Checklist and booklet considered useful. Strengths included structured flexibility and caregiver focus; limitations included late timing and limited usefulness for some subgroups. |
| Kim YJ et al. (8) | Post-treatment (4 weeks) | [H] Feasibility / Acceptability | **Implementation outcomes** - Adoption / Uptake (recruitment) = Of 119 referred, recruitment rate 15%. All 18 participants completed the study. - Feasibility (intervention delivery) = Median of 4 EC+PST sessions delivered (range 3–5). - Intervention adherence / fidelity = 100% completed sessions and homework as planned, with no major deviations. |
| Bergman M et al. (15) | Post-treatment (8 weeks), 3 months | [H] Feasibility / Acceptability | **Implementation outcomes** - Adoption / Uptake (recruitment and retention) = 91% completed treatment and post-assessment; 9% discontinued before session 4. 73% completed 3-month follow-up. No baseline differences between completers and non-completers. - Feasibility (intervention delivery) = 100% received planned sessions; no adverse events. Treatment fidelity (mean score 51/56; k = 0.80). - Acceptability (patients) = CSQ-3 mean = 10.9/12. |
| van Gils P et al. (25) | Post-treatment (6–10 weeks), 150 days | [B] Daily cognitive problems | Personal problems = weighted Tau-U = 0.189 (p < 0.01) Attention problems = weighted Tau-U = 0.181 (p = 0.02) Memory = weighted Tau-U = -0.063 (p=0.36) Keeping overview = weighted Tau-U = 0.121 (p=0.13) Planning = weighted Tau-U = 0.007 (p=0.39) |

** All outcomes were also evaluated at 3 months and no changes were observed.*

*The square bracket reports the domain to which each outcome belongs, as specified in Table 4: [B] = Cognitive outcomes; [C] = Functional status and participation; [E] = Health-related and global quality of life; [F] = cardiovascular clinical endpoints; [H] = Feasibility and implementation outcomes*

**Supplemental Table 3.** Secondary and/or exploratory outcomes and observed effect size across studies

| **Study** | **Follow-up** | **Outcome domain** | **Effect size** |
| --- | --- | --- | --- |
| **RCTs** | | | |
| Moulaert VRM et al. (14) | 1 year after CA* | [A] Post-traumatic stress | IES = mean difference ± SE = -4.18 ± 2.27 (p=0.068) |
|  |  | [A] Anxiety and depression | HADS total = mean difference ± SE = -3.25 ± 1.02 (p=0.002) HADS anxiety = mean difference ± SE = -1.79 ± 0.54 (p=0.001) HADS depression = mean difference ± SE = -1.36 ± 0.58 (p=0.020) |
|  |  | [B] Cognitive function (orientation, memory, executive function, processing speed, attention, verbal memory, cognitive lapses) | Cog-Log = mean difference ± SE = -0.08 ± 0.37 (p=0.833) AMIPB = mean difference ± SE = 0.05 ± 1.05 (p=0.961) TMT-A = mean difference ± SE = 5.13 ± 3.21 (p=0.113) TMT-B = mean difference ± SE = 10.53 ± 6.79 (p=0.124) Verbal Fluency = mean difference ± SE = -1.28 ± 0.94 (p=0.175) Paragraph Recall direct = mean difference ± SE = -0.19 ± 0.41 (p=0.633) Paragraph Recall delayed = mean difference ± SE = -0.61 ± 0.40 (p=0.130) CFQ = mean difference ± SE = -1.31 ± 1.99 (p=0.513) |
|  |  | [C] Extended daily activities | FAI = mean difference ± SE = -0.76 ± 1.05 (p=0.472) |
|  |  | [C] Return to work | Return to work = 74% (intervention) vs 71% (comparator) (p=0.734) |
|  |  | [C] Key supporter burden | CSI = mean difference ± SE = -0.12 ± 0.45 8p=0.786) |
| Cowan MJ et al. (24) | 2 years after enrolment | [F] All-cause mortality | All cause mortality = unadjusted HR = 0.38 (p=0.13) |
|  |  | [F] Cardiac events | Number of patients with a non-fatal cardiac events - Nonfatal MI (not available for 4 patients) = 1 intervention, 0 comparator (p>0.05) - Nonfatal ventricular fibrillation (not available for 4 patients) = 0 intervention, 2 comparator (p>0.05) - Nonfatal AICD/PCD firings (not availble for 4 patients) = 11 intervention, 9 comparator (p>0.05) |
|  |  | [G] Autonomic regulation | Heart rate variability = effect size not reported but p>0.05 Heart rate = effect size not reported but p>0.05 Breathing rate =effect size not reported but p=0.0001 |
|  |  | [A] General psychological distress | SCL-90-R = effect size not reported but p>0.05 |
|  |  | [H] Feasibility / Acceptability | **Implementation outcomes** - Feasibility (delivery) = 11 sessions delivered per protocol; minor scheduling deviations; <4% attrition. - Fidelity = 100% completion of protocol components and training objectives. |
| **Non RCTs** | | | |
| Moulaert VRM et al. (23) | Post-treatment (~3 months) | - | - |
| Kim YJ et al. (8) | Post-treatment (4 weeks) | [C] Self-care and daily activities | PASS-SR habit = r effect size = 0.02 (p=0.806) PASS-SR skill = r effect size = 0.04 (p=0.770) |
|  |  | [C] Instrumental activities of daily living | FAQ = r effect size = 0.01 (p=0.948) |
|  |  | [C] Objective and subjective participation | POPS objective = r effect size = 0.12 (p=0.364) POPS subjective = r effect size = 0.02 (p=0.837) |
|  |  | [D] Fatigue impact | MFIS total = r effect size = 0.24 (p<.001) MFIS physical = r effect size = 0.25 (p=0.001) MFIS cognitive = r effect size = 0.23 (p=0.006) |
|  |  | [D] Fatigue severity | FSS = r effect size = 0.11 (p=0.222) |
|  |  | [D] Patient-reported fatigue | PROMIS Fatigue scale = r effect size = 0.19 (p=0.120) |
| Bergman M et al. (15) | Post-treatment (8 weeks), 3 months | [G] Physical activity (daily steps) | Daily steps = Hedges' g = -1.2 (p=0.026) |
|  |  | [G] Sedentary behavior | Sedentary time = Hedges' g = 1.2 (p=0.02) |
|  |  | [G] Sleep patterns | Sleep duration = effect size not reported but p>0.05 |
|  |  | [A] Post-traumatic stress | 8 weeks CAPS-5 = Hedges' g = 1.59 (p<0.001) PCL-5 = Hedges' g = 1.29 (p=0.02) 3 months CAPS-5 = Hedges' g = 1.79 (p<0.001) PCL-5 = Hedges' g = 1.98 (p<0.001) |
|  |  | [A] Depression | 8 weeks HAM-D = Hedges' g = 0.61 (p>0.05) BDI-II = Hedges' g = 1.08 (p=0.006) 3 months HAM-D = Hedges' g = 1.23 (p=0.008) |
|  |  | [A] Cardiac-related anxiety | 8 weeks - ASI-3 = Hedges' g = 1.16 (p=0.004) |
|  |  | [A] Interoceptive awareness | MAIA-2 emotional awareness = Hedges' g = -1.30 (p=0.004) MAIA-2 self regulation = Hedges' g = -1.20 (p=0.005) MAIA-2 body listening = Hedges' g = -0.87 (p=0.025) |
| van Gils P et al. (25) | Post-treatment (6–10 weeks), 150 days | [B] Everyday memory failures | No effect size metrics were derived for cognitive tests and questionnaires. Participant outcomes were summarized descriptively. |
|  |  | [B] Attention / working memory |  |
|  |  | [B] Memory (objective) |  |
|  |  | [B] Subjective cognitive complaints |  |
|  |  | [B] Cognitive executive function |  |
|  |  | [B] Daily subjective cognitive complaints |  |
|  |  | [C] Participation restrictions |  |
|  |  | [E] Global life satisfaction |  |
|  |  | [H] Feasibility / Acceptability | **Implementation outcomes** - Adoption/Uptake: 100% completed the intervention and post‑treatment assessments; 80% met adherence criteria for home‑based training; 80% completed the follow‑up assessment. - Feasibility: 100% received the planned therapist‑guided sessions; the intervention was delivered as intended with only minor procedural deviations; no adverse events or drop‑outs were reported. - Acceptability: Although no formal satisfaction measures were collected, high session attendance, strong adherence in most participants, and full treatment completion. |

** All outcomes were also evaluated at 3 months, and all outcomes were unchanged except return to work, which was higher in the intervention group.*

*The square bracket reports the domain to which each outcome belongs, as specified in Table 4: [A] = Psychological and emotional outcomes; [B] = Cognitive outcomes; [C] = Functional status and participation; [D] = Fatigue; [E] = Health-related and global quality of life; [F] = cardiovascular clinical endpoints; [G] = Behavioural and psychological marker; [H] = Feasibility and implementation outcome.*
